# Supplementary material for: Detection of fish movement patterns across management unit boundaries using age-structured Bayesian hierarchical models with tag-recovery data
Source: PLoS One. 2020 Dec 7;15(12):e0243423. doi: 10.1371/journal.pone.0243423 (PMC7721192; doi:10.1371/journal.pone.0243423)
Supplement: S1 Fig — (a) Age compositions for each length class. TL = total length (mm). Solid lines and points indicate posterior median values. Ribbons and dotted lines indicate 95% credible intervals. (b) Posterior density (black solid line) and prior density (black dotted line) of the initial year correction factor from the Model AYc. Ribbon indicates the upper 95% percentile of the posterior distribution. Blue dotted line indicates posterior median value. (DOCX) [file pone.0243423.s001.docx]

**
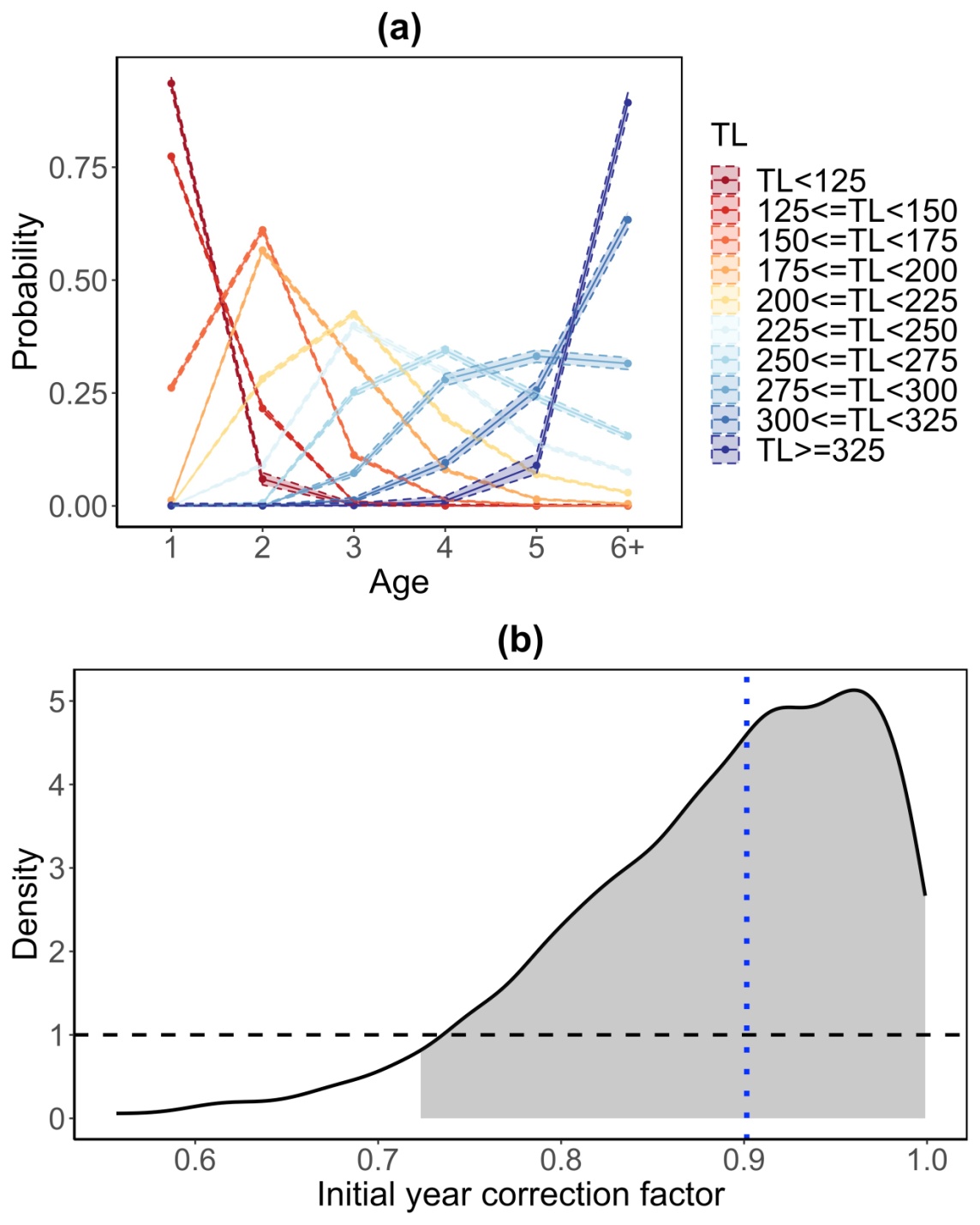
**

**S1 Fig. (a) Age compositions for each length class.** TL = total length (mm). Solid lines and points indicate posterior median values. Ribbons and dotted lines indicate 95% credible intervals. **(b) Posterior density (black solid line) and prior density (black dotted line) of the initial year correction factor from the Model AYc.** Ribbon indicates the upper 95% percentile of the posterior distribution. Blue dotted line indicates posterior median value.
