# Supplementary material for: Detection of fish movement patterns across management unit boundaries using age-structured Bayesian hierarchical models with tag-recovery data
Source: PLoS One. 2020 Dec 7;15(12):e0243423. doi: 10.1371/journal.pone.0243423 (PMC7721192; doi:10.1371/journal.pone.0243423)
Supplement: S3 Fig — In each panel, solid lines denote posterior densities, dotted line denotes prior density, and shaded areas indicate 95% credible intervals. (DOCX) [file pone.0243423.s003.docx]

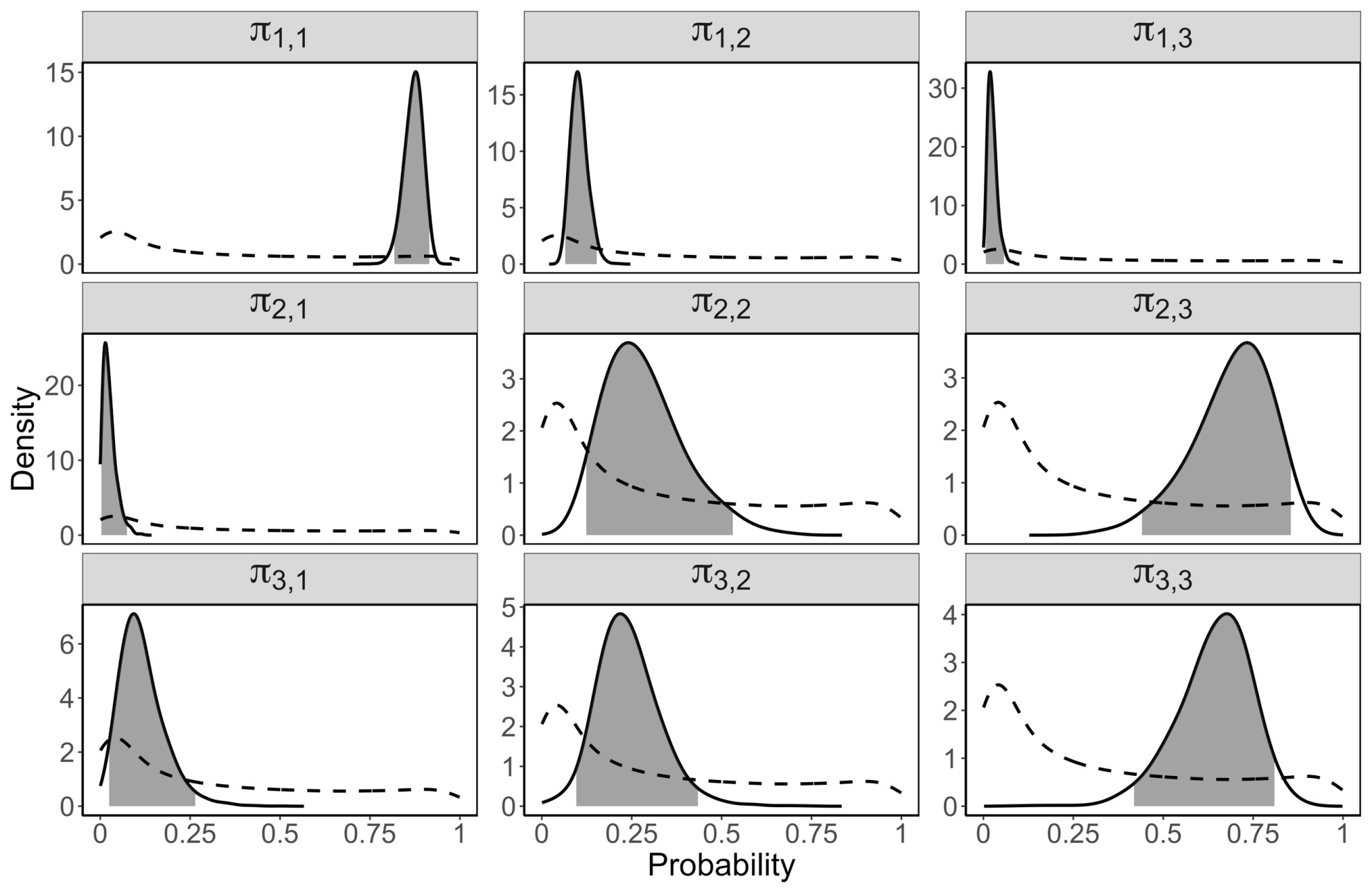


**S3 Fig. Movement probability of a tagged yellow perch from Model C.** In each panel, solid lines denote posterior densities, dotted line denotes prior density, and shaded areas indicate 95% credible intervals.
