# Supplementary material for: Detection of fish movement patterns across management unit boundaries using age-structured Bayesian hierarchical models with tag-recovery data
Source: PLoS One. 2020 Dec 7;15(12):e0243423. doi: 10.1371/journal.pone.0243423 (PMC7721192; doi:10.1371/journal.pone.0243423)
Supplement: S4 Fig — (a) Posterior density of natural mortality. The blue dotted line indicates posterior median. The ribbon indicates 95% credible interval. (b) Posterior estimates of age-specific selectivity. The solid lines and points indicate posterior median values. The ribbons indicate 95% credible intervals. (c) Posterior estimates of year-specific commercial fishing mortality within each MU. The solid lines and points indicate posterior median values. The ribbons indicate 95% credible intervals. Point estimates from the YPTG stock assessment model for each region were denoted by asterisks. (DOCX) [file pone.0243423.s004.docx]

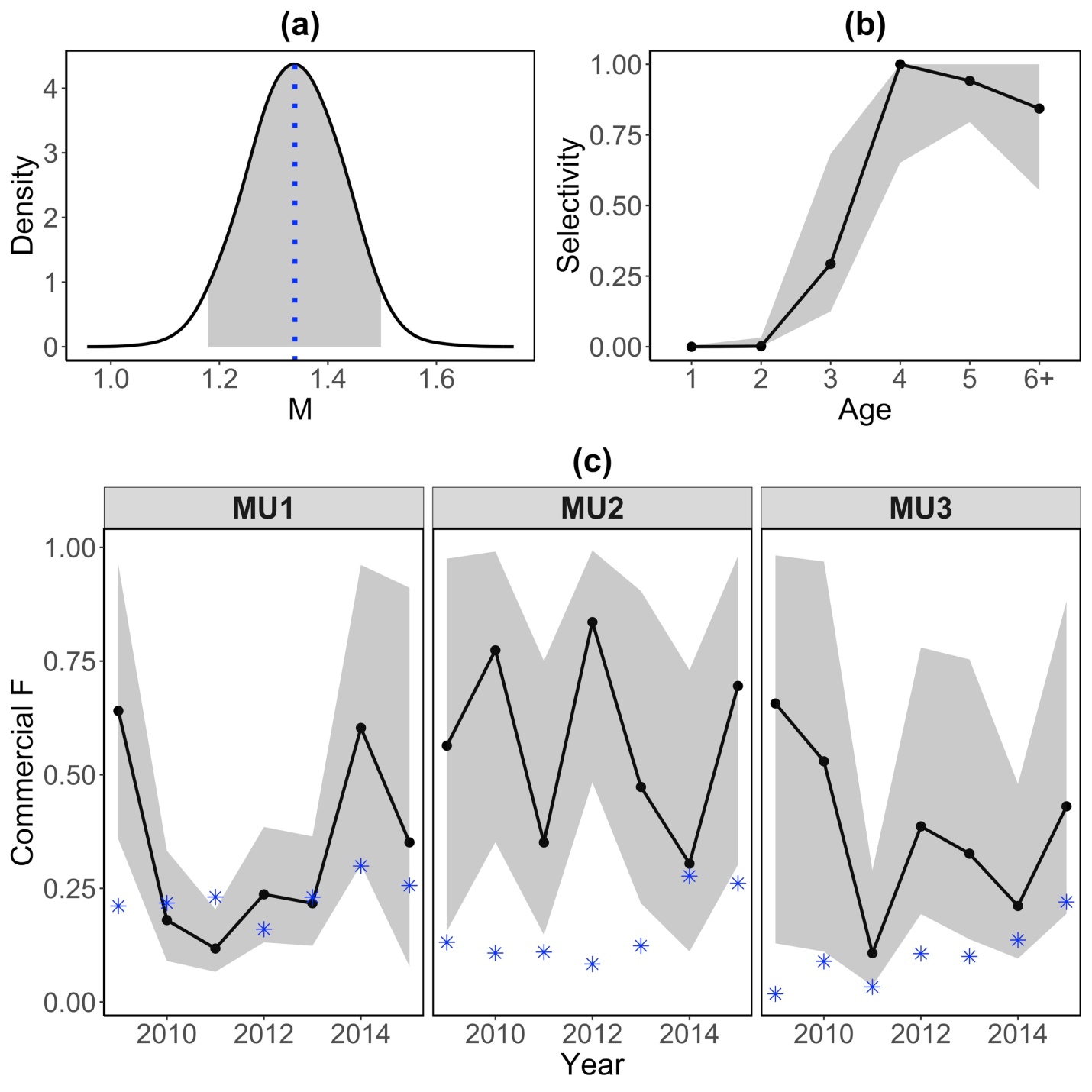


**S4 Fig. Estimations of additional biological and fishery-related parameters from Model AR.** (a) Posterior density of natural mortality. The blue dotted line indicates posterior median. The ribbon indicates 95% credible interval. (b) Posterior estimates of age-specific selectivity. The solid lines and points indicate posterior median values. The ribbons indicate 95% credible intervals. (c) Posterior estimates of year-specific commercial fishing mortality within each MU. The solid lines and points indicate posterior median values. The ribbons indicate 95% credible intervals. Point estimates from the YPTG stock assessment model for each region were denoted by asterisks.
