# Supplementary material for: Detection of fish movement patterns across management unit boundaries using age-structured Bayesian hierarchical models with tag-recovery data
Source: PLoS One. 2020 Dec 7;15(12):e0243423. doi: 10.1371/journal.pone.0243423 (PMC7721192; doi:10.1371/journal.pone.0243423)
Supplement: S6 Fig — (DOCX) [file pone.0243423.s006.docx]

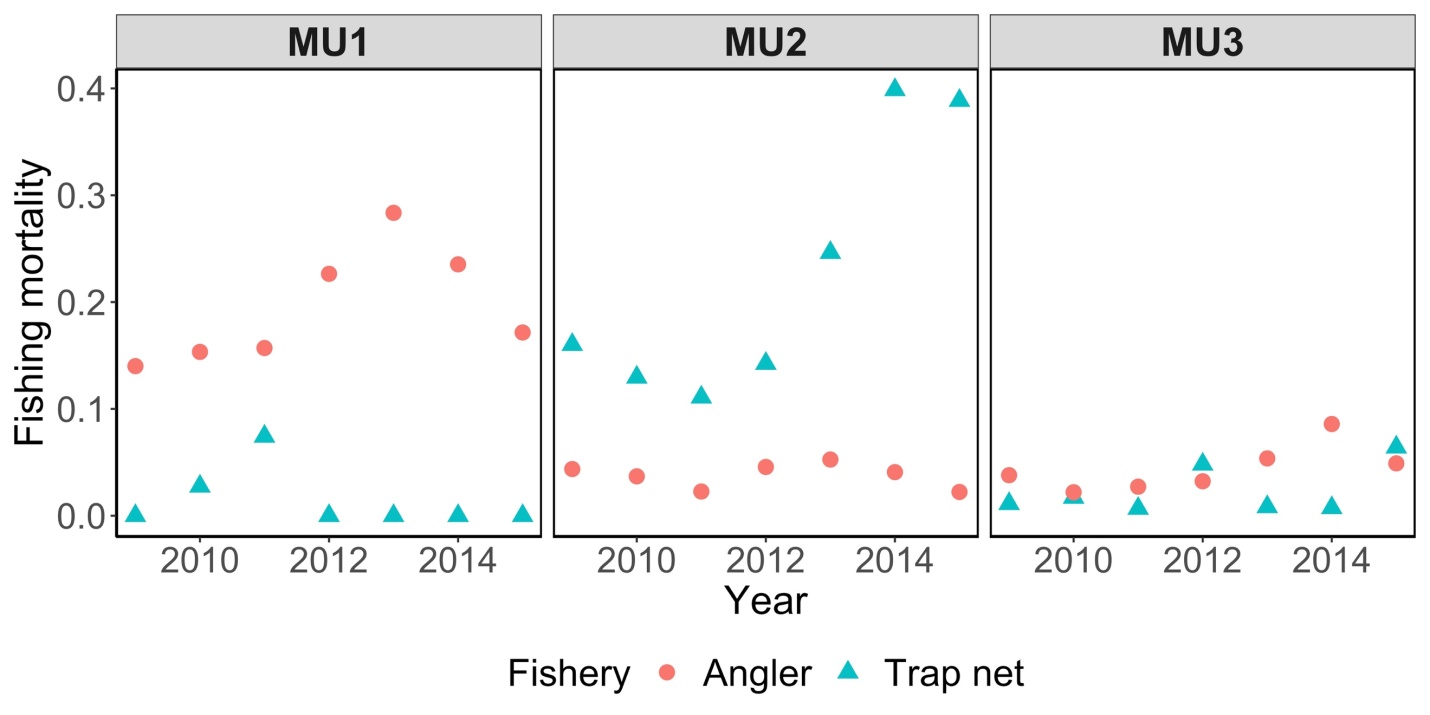


**S6 Fig.** **Point estimates of commercial trap net and angler fishing mortality rates in the U.S. waters estimated from the YPTG stock assessment model.**
