# Supplementary material for: Detection of fish movement patterns across management unit boundaries using age-structured Bayesian hierarchical models with tag-recovery data
Source: PLoS One. 2020 Dec 7;15(12):e0243423. doi: 10.1371/journal.pone.0243423 (PMC7721192; doi:10.1371/journal.pone.0243423)
Supplement: S1 Table — Bold symbols represent vectors and matrices, and regular symbols represent scalars. (DOCX) [file pone.0243423.s007.docx]

**S1 Table. Symbols used in the model equations.** Bold symbols represent vectors and matrices, and regular symbols represent scalars.

| **Symbol** | **Description** |
| --- | --- |
|  | **Indicator variables** |
| $a$ | Age (1 – 6+) |
| $k$ | Region (1 – 3) |
| $tk$ | Tagging region |
| $fk$ | Recovery region |
| $y$ | Year (2009 – 2015) |
| $ty$ | Tagging year |
| $fy$ | Recovery year |
| $l$ | Length class |
|  | **Fixed quantity** |
|  | *Partnership gillnet surveys data* |
| $n_{l}$ | Age-specific abundance of length class $l$ |
| $N_{l}$ | Sample size for length class $l$ |
|  | *Scanning rates in Ontario commercial fishery* |
| $\boldsymbol{\lambda}$ | Matrix whose ($fy,fk$)*^th^* element $\lambda_{fy,fk}$ is the tag reporting rate in year $fy$ from region $fk$ |
|  | **Estimated parameters** |
| $\boldsymbol{\pi}$ | Array whose ($k,k^{'},a,y$)*^th^* element $\pi_{k,k^{'},a,y}$ is the movement probability that a tagged age-$a$ fish moves from region $k$ to region $k^{'}$ in year $y$ |
| $\boldsymbol{u}$ | Array whose ($a,fy,k$)*^th^* element $u_{a,fy,k}$ is the exploitation rate of a tagged age-$a$ fish in year $fy$ from region $k$ |
| $\boldsymbol{S}$ | Array whose ($a,y,k$)*^th^* element $S_{a,y,k}$ is the survival rate of a tagged age-$a$ fish in year $y$ in region $k$ |
| $\boldsymbol{s}$ | Vector whose $a$*^th^* element $s_{a}$ is the selectivity of commercial gillnet fishery on age-$a$ fish |
| $\boldsymbol{F}$ | Matric whose ($k,fy$)*^th^* element $F_{k,fy}$ is the commercial gillnet fishing mortality in region $k$ in year $fy$ |
| $M$ | Instantaneous natural mortality |
| $ratio$ | Initial year adjustment factor |
| $\boldsymbol{\kappa}_{l}$ | Age composition of length class $l$ |
| $\boldsymbol{P}_{a,ty,tk,fy}$ | Vector whose $fk$th element $P_{a,ty,tk,fy,fk}$ is the probability of a fish at age $a$ tagged in year $ty$ in region $tk$ being recovered in year $fy$ from region $fk$ |
|  | **Hyperparameters** |
| $\eta_{1}$ | Inflection point of the increasing logistic curve |
| $\eta_{2}$ | Slope of the increasing logistic curve |
| $\eta_{3}$ | Inflection point of the decreasing logistic curve |
| $\eta_{4}$ | Slope of the decreasing logistic curve |
| $\gamma_{k,k^{'}}$ | Movement parameter from region $k$ to region $k^{'}$ |
|  | *Movements with random effects by year* |
| $\psi_{k,k’}$ | Hyperparameters of movement parameters |
| $\tau$ | Variance of year effect on movement parameter |
|  | *Movements with random effects by age* |
| $\phi_{k,k’}$ | Hyperparameters of movement parameters |
| $\omega$ | Variance of age effect on movement parameter |
|  | *Movements with random effects by year and age* |
| $\varphi_{k,k’}$ | Hyperparameters of movement parameters |
| $U_{y}$ | Year effect on movement parameter |
| $U_{a}$ | Age effect on movement parameter |
| $\nu_{y}$ | Variance of year effect on movement parameter |
| $\nu_{a}$ | Variance of age effect on movement parameter |
