# Supplementary material for: Detection of fish movement patterns across management unit boundaries using age-structured Bayesian hierarchical models with tag-recovery data
Source: PLoS One. 2020 Dec 7;15(12):e0243423. doi: 10.1371/journal.pone.0243423 (PMC7721192; doi:10.1371/journal.pone.0243423)
Supplement: Attachment — Submitted filename: yellowperch_review.pdf [file pone.0243423.s008.pdf]

Overview
Bi et al. present a modeling framework for estimating age/length-based movement of fish between discrete management units. They apply their framework to a large tag-recovery dataset on yellow perch in Lake Erie, and find that the model with random effects of age and year on movement rates is most supported. Their model was able to estimate natural mortality, exploitation rate, and selectivity in addition to the probabilities of movement between three regions.

Impression
I found the modeling approach adequately motivated and sound, and I believe it will be useful in other situations. I have minor questions about the novelty of the model framework and a couple parameterization choices. The conclusions are generally supported by the data, although the year and age effects on movement rates are not directly presented and may be overstated. Overall, I imagine that these concerns can be addressed via major revision, and the manuscript warrants eventual publication.

Primary concerns
The novelty of the model is not made clear in the introduction and methods. Surely age-structured mark-recapture models with effects of age and year on movement rates have been published previously, yet they are not referenced in relation to the model presented. How does this model differ from others? Does it? There is a rich literature on mark-recapture models that is not adequately referenced. The one reference given in the methods about “this type of model” is incorrect—the cited paper uses a different type of data (continuous tracks from archival tags) and does not estimate a parametric model at all.

I recognize that the parameters were estimable and the models converged. Thus, the following may be addressable in the text, without model modification. Yet, two modeling choices seemed questionable:

1. Treatment of natural mortality.
   a) The authors estimate M as $0.4e^{\delta}$, where $\delta \sim N(0,\nu)$ and $\nu \sim U(0,0.1)$. Their estimate of M is 1.3. This means that even if ν is at the upper bound of its prior, $\delta = \log(1.3/0.4) = 1.18$, which is 11.8 standard deviations from the mean of 0. There is no justification given for the choice of $\nu \sim U(0,0.1)$, yet if it is at the upper bound, it may influence the value of M. Supplemental figures with prior/posterior plots of δ and ν would convince readers that M is well-estimated.
   b) M is constant across ages, so if a large (0.9/1.3) proportion of M is actually fishing mortality from U.S. waters, your model is vastly overestimating mortality of young (pre-selected) fish. This at least needs to be discussed, if not addressed directly in the model.
2. Fixing one movement parameter per region such that the probabilities sum to one is an odd choice. Why not use a likelihood designed for compositions, e.g. multinomial, Dirichlet, Dirichlet-multinomial, or logistic normal?

The year and age effects on movement rates are reported as “substantial”, but they are not directly presented and may be overstated. The final model included random effects of year, U_y, and age, U_a. Yet the U_y and U_a from the final model were never presented with 95% CIs, either in the text or figures. Figures 2-5 only show modest variation in movement rates, for few ages and years.

The text could use more editing to improve clarity and precision. There are grammatical errors and vague statements, although for the most part they do not prevent the methods or results from being understood. Frequently, the same amount of information could be conveyed with fewer words, e.g. “non-intrusive auxiliary measurements of tagged individuals, such as total length information, are recorded...” could be simplified to “length data can be recorded non-intrusively...” (lines 57-58).

Section-specific comments

Abstract

18-19: I am not convinced that the age and year effects were substantial. The final model included random effects of year, U_y, and age, U_a. Yet the U_y and U_a from the final model were never presented with 95% CIs, either in the text or figures. The posteriors in Figs. 2-4 are barely different by year or age.

Introduction

26-27: The importance of individual movement seems overstated. “can have profound” instead of “profound”.
31-33: You imply that limited data is the only reason stock assessments don’t include spatial structure, which is not true. Individual movement data may not actually be that important at the population/stock level, beyond informing stock structure. Fitting individual movement data in assessments also complicates the model code and increases runtimes.
34-37: Unique marks can also be used to ID individuals in fish. Also, when individual ID is used, the resulting data is capture/mark-recapture (not sure the distinction is necessary).
42: I’d distinguish telemetry by saying it can provide a continuous location track as opposed to discrete start/stop endpoints.
34-44: Too vague. Should introduce PIT tags, as they’re what this study used, in relation to other types of tagging.
48: Uncertainties? Parameters to estimate
54-57: Could at least mention nonlethal aging is possible, e.g. of fin rays: https://peerj.com/articles/1010/
57-58, “non-intrusive auxiliary measurements of tagged individuals, such as total length information, are recorded...” could be simplified to “length data can be recorded non-intrusively...”
61: Age structure, not population structure

Methods

104-110: How likely is it to go from 1 to 3 without going through 2 (i.e. through US waters)?
113-114: Ref 33 does not use comparable data or apply a similar model. Block et al. (2005) used tags that record continuous tracks and created kernel density estimates for groups of fish (W/E spawners, size bins) to estimate probability of remaining in the west region or moving to the east. It’s not even a parametric model! Please cite another reference(s) where an age-structured tag-return model is used.
119: Clarify that pi are probabilities of movement
123: Can you add any justification for this assumption? PIT tag retention rates are often high, so this is reasonable. I’d just add a reference, as specific to yellow perch and injection method (muscle, ventral) as possible.
124: Clarify what is in Table 2: proportion of commercial catch sampled in region k and year fy. “Derived from”, or “equal to”?
A table listing the symbols in the model, clarifiying which are estimated parameters versus fixed quantities, would be useful. For example, I believe the lambdas were calculated as in Table 2, not estimated.
144: “expected to be greater than 0.4”
144-149: Curious why you didn’t restrict delta to be non-negative, since you have a strong expectation that M should not be less than 0.4. Although I see delta was estimated to be positive, so perhaps it doesn’t matter.
155-161: This seems odd. Instead of fixing 1 of 3 probabilities per region, why not use a likelihood for compositional data/parameters? Examples: multinomial, Dirichlet, Dirichlet-multinomial, logistic

normal. Hyperpriors can easily be specified to make the prior probability of not moving slightly higher than moving.
182: Would be useful to describe YR as “random effects by year”, as opposed to the fixed effects described on 175. Also would be useful to give a motivation, e.g. this reduces the effective number of parameters while still allowing movement to vary by year.
195-199: Same as above.
237-238: Instead of “relatively large samples”, please report the actual sample sizes
246-260: This section is titled “Likelihood” yet there is no data or likelihood described where the Pr(recap) and Pr(at large) are used. You probably meant to add a binomial or Bernoulli here…
261: Suggest “Model estimation” for the subtitle.
267: I think using DIC for model selection is fine, but WAIC or LOO are generally preferred and can be calculated in JAGS models without too much extra work: https://link.springer.com/article/10.1007/s11222-016-9696-4.

Results
275-277: Calling the estimated year and age effects on movement rates “substantial” is a stretch. If you added CIs to Fig. 5 my guess is there would be quite a bit of overlap. The posteriors in in Figs. 2-4 are almost completely overlapping with no discernible differences apart from the two examples you highlight later (2012 more 3 -> 3, 2011 more 2 -> 3). I think this sentence should be dropped or modified.
277-281: Using random effects vs. fixed effects terminology consistently to describe the difference between models YR vs. YS and AR vs. AS would make the paper clearer.
297-300: Suggest adding that most year and age effects were not pronounced, except for the two examples you highlight.
312-322: Could be summarized more briefly—across all ages and years most yellow perch in MU1 stayed in MU1. 316-318 can be cut, does not add any new information.
343-343: M = 1.3 implies that delta = 1.18. If nu = 0.1, your estimate of delta is 11.8 standard deviations from the mean.

Discussion
374-377: Should report the estimate of M with 95% CI here, instead of just stating it was greater than 0.4. Also, M = 1.3 is quite a bit higher than 0.4. Are estimates of F from bycatch or U.S. harvest available to account for a substantial portion of this difference? Otherwise, that is a lot of emigration, tag loss, and/or tagging mortality.
378: I’m not sure that additional information *needs* to be collected to account for these factors… your model is useful as is. Additional information *could be* collected.
397-398: But this was true in all years, not just 2012.
428: Region was not a random effect in the model

Figures
Not absolutely necessary, but the multi-panel figures 2-5 and 7 would look better with reduced margins and shared axes.
Figs. 2-5: Because the year and age effects are additive (the effect of year is the same across ages), I think these could be improved by displaying one set focused mean movement (comparing priors and posteriors), one set on year effects (i.e. Fig. 5, removing ages and adding credible intervals instead), and one set focused on age effects (i.e. Figs. 2-4, removing years). CIs should be added so the reader can see the degree of age and year effects.
Fig. 5 has different y-axes for each panel. Keeping the y-axes consistent from 0-1 would make it easier to digest where the fish are moving.

Figs. 5 and 7 do not have estimates of uncertainty, and therefore it is impossible to know what degree the annual variations are significant. CIs should be added.
Fig. S1: A gradient color scale may make this easier to digest (i.e. adjacent length bins should be more similar in color).
