# Supplementary material for: Detection of fish movement patterns across management unit boundaries using age-structured Bayesian hierarchical models with tag-recovery data
Source: PLoS One. 2020 Dec 7;15(12):e0243423. doi: 10.1371/journal.pone.0243423 (PMC7721192; doi:10.1371/journal.pone.0243423)
Supplement: Attachment — Submitted filename: Response to Reviewers.docx [file pone.0243423.s009.docx]

Dear editor and reviewers,

Thank you for your helpful comments. We have revised our paper accordingly and feel that your comments helped clarify and improve our paper. Please find our responses (in blue) to reviewer’s specific comments (in black) below.

Respectful,

Rujia

Reviewer #1: General issues:

The idea behind the study and the chosen modeling approach are reasonable. The use of random effect to model temporal and age-specific variation in movement rates is innovative and make this manuscript of potentially widespread interest. However, large gaps in the methods and mismatches between the equations and the stated results in multiple areas (noted below) suggest serious flaws in the write-up and potentially in the results as well.

Neither Table 1 nor Figure 1 provide any indication of what fraction of recaptures in the three management areas originated which of the two release areas so it’s impossible to assess how reasonable the estimate movement rates are. There is also no discussion of how reasonable it is to estimate the large number of parameters and random effects relative the quantity of data available: 322 recaptured fish are used to estimate 2 movement parameters for each of 3 areas for 7 years and 6 ages = 252 independent quantities (as shown in Figure 5). The use of random effects means that the true number of parameters is much lower, but the precision the interpretation of the time-varying quantities and any consideration of links to environmental variables needs to acknowledge the potential overparameterization of this model. Of particular concern is the estimates of annual and age-specific changes in movement rates originating in MU3 when there were no tags released in MU3. However, with no likelihood equation provided it is not possible to evaluate whether the chosen approach was appropriate (such as through the use of the negative binomial likelihood which includes an overdispersion parameter, which is a common approach in the analysis of fisheries tagging data).
We revised Table 1 to provide more details on tag and recovery region. We provided likelihood equation (Equation 26), and each recovered and not recovered fish contributed to the likelihood. We found that “Uncertainties on movement probabilities $\pi_{2,2}$, $\pi_{2,3}$, $\pi_{3,1}$, $\pi_{3,2}$ and $\pi_{3,3}$ were larger owing to less fish tagged and released from MU2 and no fish tagged and released from MU3 (Table 1), as well as lower tag reporting rate in MU3 (Table 2). The posterior densities for $\pi_{3,1}$ were little changed compared with the prior density (Fig 2c), due to limited information as stated above.”

Model with random year and age effects (Model AY), model with random age effects (Model AR) and model with constant movements (Model C) all revealed the same movement patterns. Model AY performed best with the smallest DIC, WAIC and LOO values (Table 3), so it was recommended. Although year and age-variations in movement probabilities derived from Model AY (Figs 3 and 4) were not substantial, there were obvious differences on posterior medians in some years and age classes, for example, posterior medians of movement probability $\pi_{3,3}$ in 2012 and 2014 were 0.65 and 0.63 respectively that were 3-9% greater than $\pi_{3,3}$ in other years; posterior median of movement probability $\pi_{1,2}$ at age-4 was 0.13 that was 30-184% greater than $\pi_{1,2}$ at the rest age classes; posterior median of movement probability $\pi_{3,2}$ at age-4 was 0.33 that was 29-58% greater than $\pi_{3,2}$ at the rest age classes.

Environmental variables such as winter NAO, ice cover, were not incorporated into the model. Regression analyses between environmental variables and time-varying movements revealed that water level displayed a significant positive impact on posterior medians of year-varied age-average movement probability from MU2 to MU3, without other statistically significant relationships found. Some links between environmental variables and time-varying movements were qualitative given the limited spatial and temporal coverage of the current experiment, and more intensive tagging experiments are needed for an in-depth analysis.

Based on model comparison criterions and same results on movement probabilities among Models AY, AR and C, we didn’t recognize overparameterization of Model AY. Model sparsity was already taken into account in the various model selection criteria used in this study to evaluate model performance.

Finally, there is no comparison of the results of this study to the Yellow Perch Task Group stock assessment reports. A visual comparison of the estimated time series of Commercial F shown in Figure 6c with the time series of fishing effort shown in YPTG reports shows little correlation.

We added point estimates of commercial gillnet F from the YPTG stock assessment report to the figure, which were denoted by asterisks. “Point fishing morality estimates from stock assessment was within the 95% credible intervals derived from Model AY for MU1 from 2010 to 2015, for MU2 in 2014 and 2015, and for MU3 in 2014 and 2015, which had more fish tagged and released. Larger sample size would be required for tagging study to derive more accurate results. The current stock assessment ignored fish movement across MUs, which also potentially led to differences in fishing mortality estimates.”

Specific issues:

Abstract: Reference to specific management units is not meaningful for anyone unfamiliar with the details of this fishery so should be made more general.

We changed to “Different movement configurations modeling the tag-recovery data were compared, and all configurations revealed that yellow perch individuals in the western basin (MU1) exhibited relatively strong site fidelity, and individuals from the central basin (MU2 and MU3) moved within this basin, but their movements to the western basin (MU1) appeared small.”

Line 55: "ageing" is a more standard spelling for this process than "aging"

Corrected.

Line 104: the "scarcity of tags recovered from U.S. waters" should be clarified. What was the scanning effort in U.S. waters (relative to what is shown in Table 2)? And how many tags were recovered? Likewise, was there any scanning effort in MU4?

We added a sentence to clarify it: “Ohio started scanning from 2013 to 2015, and got 10 yellow perch that were tagged in Ontario”. Because the US side's coverage was limited both in space (only Ohio) and in time (13-15), it was not included in the model. Scanning rates in MU4 was added to Table 2.

Line 116: Equations would benefit from numbers to make them easier to reference.

Done.

Line 147: This equation does not make sense if M is believed to be greater than 0.4 as the expectation is equal to 0.4. Also, why is M modeled hierarchically? It appears there is a single M applied to all ages and areas (implied by figure 6a) so a prior applied directly to M makes more sense than this structure. Finally the posterior distribution in Figure 6a can’t have come from this equation, as even with nu at the upper bound of 0.1, the likelihood of a value of M = 1.3 is infinitesimally small.

We had set nu over the interval (0, 1) instead of (0, 0.1), which was a mistake in writing. In this revision and based on the reviewer suggestion, we modeled M with a uniform prior over the interval (0.2, 2).

Line 156: "the movement parameter for not moving … was fixed to a prescribed constant". The chosen constant should be included in the text.

We had chosen a constant of log(4) to be used in the random year and random age (AY) model, log(3) in the random age (AR) model and random year (YR) model, and log(2) in the constant (C) model. In this revision, we set weakly informative Dirichlet priors with elemental gamma hyperprior gamma(1,1) for the three movement probabilities per region as suggested by reviewers.

Line 157: the interval for the uniform U(-1, 1) prior on the movement parameters is too restrictive. For instance, if unspecified constant related to not moving was set to 0, then the probability of movement to any other area would be limited to the range from exp(0)/(exp(0) + exp(-1) + exp(-1)) = 0.21 to exp(0)/(exp(0) + exp(1) + exp(1)) = 0.42. Higher and lower values of the unspecified constant would shift this range to higher or lower values but will always span less than half of the 0 to 1 range I would have expected to be available for consideration by the model. If there is external information to develop an informative prior, then a uniform distribution seems like a poor choice. Although the calculations above don’t include the addition of the random effects, I don’t see how the distributions shown in Figures 2-4 could correspond to the model as described in this section.

Please see our response above.

Line 170: This equation is the same as the one on line 154 (which is presumably presented as a general form representative of all scenarios). In this case the previous equation could simply be referenced.

Done.

Line 245: This section with heading "Likelihood" doesn’t include a likelihood equation, only the expected probabilities of a tag being recaptured or remaining at large. The likelihood would compare these expected values with the observations.

We added description on likelihood function. “The tag recoveries by year of release (𝑡𝑦), region of release (𝑡𝑘), year of recovery (*fy*), region of recovery (*fk*) and length class at release (*l*) and tags that were never recovered were assumed to be multinomially distributed.” The likelihood function was provided in Equation 26.

Line 283: Caption should define all columns.

Done.

Line 264: A citation for the R software be added, as provided by the R function "citation()".

Done.

Line 312: "in that region" or similar phrase is missing from the sentence.

Corrected.

Line 320: Discussion of age-4 as having a higher movement rate from MU1 to MU2 compared to other ages doesn’t align well with Figure 5 which shows very similar rates between most ages.

Please see our new Fig 4 that showing age-varied year-average movement probability. Age-4 individuals were more likely to move outwards – from MU1 to MU2 (Fig 4a), from MU2 to MU3 (Fig 4b), from MU3 to MU2 (Fig 4c). Posterior median of movement probability $\pi_{1,2}$ at age-4 was 0.13 that was 30-184% greater than $\pi_{1,2}$ at the rest age classes; posterior median of movement probability $\pi_{2,3}$ at age-4 was 0.72 that was 2-10% greater than $\pi_{2,3}$ at the rest age classes; posterior median of movement probability $\pi_{3,2}$ at age-4 was 0.33 that was 29-58% greater than $\pi_{3,2}$ at the rest age classes.

Lines 332-340: It should be acknowledged that there were zero releases in MU3. In this context you would expect the posterior estimates for movement out of MU3 to closely match the post-model pre-data ("prior") distributions as they do.

We acknowledged that the posterior densities for $\pi_{3,1}$ were little changed compared with the prior density (Fig 2c), due to limited information provided by the zero releases in MU3.

Line 357: "the spatially structured tagging model framework is relatively new" is contradicted by the reference later in the paragraph to the Maunder (1998) dissertation and the earlier citation of Hilborn (1990). I agree that there have been relatively few such models applied to real-world data to directly estimate movement rates. Another example worth including would be Punt, A.E., Pribac, F., Walker, T.I., Taylor, B.L. and Prince, J.D., 2000. Stock assessment of school shark, Galeorhinus galeus, based on a spatially explicit population dynamics model. Mar. Fresh. Res., 51:205-220.

We changed the reference cited, and add Punt et al. 2000.

Figure 1: The figure would benefit from either lines connecting releases to recaptures, colors indicating source for recaptures, and/or some indication of the sample sizes associated with the recapture positions. See mark-recapture literature for examples.

We replotted Fig 1 with difference colors and symbols indicating tagging locations and sources for recaptures.

Figures 2-4: The "prior density" represented in these figures needs to be explained in the methods. This is presumably a post-model pre-data distribution associated with the movement rates based on the priors on the associated parameters. But since the resulting movement rate is not a parameter, but a quantity derived from a combination of parameters, this is not a prior. Also, I would use "shaded area" rather than "ribbons" to described the element which indicates 95% credibility interval. I think "ribbon" makes more sense for a time series plot like 6c and is reasonable for 6b as well.

We defined “ prior density” in Movement configuration subsection under Methods section. “Ribbons” related to 95% credible intervals was changed to “shaded areas”.

Figure 5: Why is the posterior mean rather than the posterior median used here?

We changed posterior mean to posterior median in this revision.

Reviewer #2

**Overview**

Bi et al. present a modeling framework for estimating age/length-based movement of fish between discrete management units. They apply their framework to a large tag-recovery dataset on yellow perch in Lake Erie, and find that the model with random effects of age and year on movement rates is most supported. Their model was able to estimate natural mortality, exploitation rate, and selectivity in addition to the probabilities of movement between three regions.

**Impression**

I found the modeling approach adequately motivated and sound, and I believe it will be useful in other situations. I have minor questions about the novelty of the model framework and a couple parameterization choices. The conclusions are generally supported by the data, although the year and age effects on movement rates are not directly presented and may be overstated. Overall, I imagine that these concerns can be addressed via major revision, and the manuscript warrants eventual publication.

Thanks for the comments. In this revision, we presented year and age effects on movement rates ($U_{y}$ and $U_{a}$) in S Figs 4 and 5 in Supporting Information

**Primary concerns**

The novelty of the model is not made clear in the introduction and methods. Surely age-structured mark-recapture models with effects of age and year on movement rates have been published previously, yet they are not referenced in relation to the model presented. How does this model differ from others? Does it? There is a rich literature on mark-recapture models that is not adequately referenced. The one reference given in the methods about “this type of model” is incorrect—the cited paper uses a different type of data (continuous tracks from archival tags) and does not estimate a parametric model at all.

In this revision, we cited more literature on spatial tag-return models in Introduction section: “Analysis of tagging data to estimate individual movement has received considerable attention in fisheries research [e.g. 16,20,21]. A spatial extension of the traditional Brownie model [22] have been widely used to derive movement probability from tagging data [20,21]. The spatial Brownie models separate parameters for survival and movement rates, and parameterize survival and recovery rates in terms of instantaneous natural morality and fishing mortality rates [20,21,23]. Interactions between individual movement and age class, time, and region make the movement process complex and difficult to understand [24]. These parameters can be included in the process model as random factors treated as parameters to estimate, so individual movement can be age-, year-, and region-dependent and modeled by formulating hierarchical structures [20,21].”

Then, we talked about the novelty of our models: “Most existing published tagging models assume that ages at tagging determined from length or from direct ageing of hard parts are known without error [e.g. 20,21]. When ages are determined from scales, biases are not expected; however, when ages are determined from length, biases can exist [20]. The sample size used to derive age-length frequency has an influence on the accuracy of the age-length relationship [27,28]; the uncertainty in age structure owing to age-length data needs to be considered.”

We corrected the references for “this type of model”: (1) Eveson JP, Laslett GM, Polacheck T. A spatial model for estimating mortality rates, abundance and movement probabilities from fishery tag-recovery data. In: Thomson DL, Cooch EG, Conroy MJ, editors. Modeling Demographic Processes in Marked Populations. Boston: Springer; 2009. pp. 987-1010. (2) Vandergoot CS, Brenden TO. Spatially varying population demographics and fishery characteristics of Lake Erie walleyes inferred from a long-term tag recovery study. Trans Am Fish Soc. 2014; 143: 188-204. (3) Li Y. Innovative stock assessment methods and management solutions for spatially structured fish populations. Ph.D. dissertation, Michigan State University. 2018.

I recognize that the parameters were estimable and the models converged. Thus, the following may be addressable in the text, without model modification. Yet, two modeling choices seemed questionable:

1. Treatment of natural mortality.
a) The authors estimate M as 0.4eδ, where δ ~ N(0,ν) and ν ~ U(0,0.1). Their estimate of M is 1.3. This means that even if ν is at the upper bound of its prior, δ = log(1.3/0.4) = 1.18, which is 11.8 standard deviations from the mean of 0. There is no justification given for the choice of ν ~ U(0,0.1), yet if it is at the upper bound, it may influence the value of M. Supplemental figures with prior/posterior plots of δ and ν would convince readers that M is well-estimated. We had set ν ~ U(0, 1) instead of U(0, 0.1), which was a mistake in writing. In this revision, we modeled M with a uniform prior over the interval (0.2, 2). Because we realized that there is a single M applied to all ages and areas so no need to model M hierarchically.
b) M is constant across ages, so if a large (0.9/1.3) proportion of M is actually fishing mortality from U.S. waters, your model is vastly overestimating mortality of young (pre-selected) fish. This at least needs to be discussed, if not addressed directly in the model.

We added a discussion on overestimating mortality of young (pre-selected) fish. “Point estimates of commercial trap net and angler fishing mortality rates in the U.S. waters estimated from the YPTG stock assessment model [49] are plotted in S Fig 6 in Supporting Information. The sum of these two fishing mortality rates is large in MU2 in 2014 and 2015 (> 0.4). If a large proportion of the estimated natural mortality is actually fishing mortality from U.S. waters, our models is overestimating the natural mortality of young (pre-selected) fish.”

2. Fixing one movement parameter per region such that the probabilities sum to one is an odd choice. Why not use a likelihood designed for compositions, e.g. multinomial, Dirichlet, Dirichlet-multinomial, or logistic normal?

In this revision, we set weakly informative Dirichlet priors with elemental gamma random variables gamma(1,1) for the three movement probabilities per region.

The year and age effects on movement rates are reported as “substantial”, but they are not directly presented and may be overstated. The final model included random effects of year, Uy, and age, Ua. Yet the Uy and Ua from the final model were never presented with 95% CIs, either in the text or figures. Figures 2-5 only show modest variation in movement rates, for few ages and years.

We plotted year-varied age-average movement probabilities in Fig 3 and age-varied year-average movement probabilities in Fig 4. Although year and age-variations were not substantial, Model AY performed best with the smallest DIC, WAIC and LOO values.

The models of constant and random age are of management purposes, especially when the tagging experiment is short-term (7 years) compared with long-term catch and survey data (45 years.  So, we made some comparisons with these models, which could further validate Model AY. All the three models revealed that yellow perch individuals in MU1 exhibited relatively strong site fidelity, and individuals from MUs 2 and 3 were likely to move between these units, but movement from MUs 2 and 3 to MU1 appeared limited.

The text could use more editing to improve clarity and precision. There are grammatical errors and vague statements, although for the most part they do not prevent the methods or results from being understood. Frequently, the same amount of information could be conveyed with fewer words, e.g. “non-intrusive auxiliary measurements of tagged individuals, such as total length information, are recorded...” could be simplified to “length data can be recorded non-intrusively...” (lines 57-58).

We edited text to improve clarity and precision in this revision.

**Section-specific comments**

*Abstract*

18-19: I am not convinced that the age and year effects were substantial. The final model included random effects of year, U_y, and age, U_a. Yet the U_y and U_a from the final model were never presented with 95% CIs, either in the text or figures. The posteriors in Figs. 2-4 are barely different by year or age.

We rewrote this part to avoid using commenting word of “substantial” variations in movement rates among years and ages. We plotted year-varied age-average movement probabilities in Fig 3 and age-varied year-average movement probabilities in Fig 4.

*Introduction*

26-27: The importance of individual movement seems overstated. “can have profound” instead of “profound”.

Done.

31-33: You imply that limited data is the only reason stock assessments don’t include spatial structure, which is not true. Individual movement data may not actually be that important at the population/stock level, beyond informing stock structure. Fitting individual movement data in assessments also complicates the model code and increases runtimes.

We revised this section, by 1) pointing out that the risks from ignoring individual movements across local populations in fisheries management; 2) pointing out the other impediment - computing power.

34-37: Unique marks can also be used to ID individuals in fish. Also, when individual ID is used, the resulting data is capture/mark-recapture (not sure the distinction is necessary).

Thanks. We revised this section.

42: I’d distinguish telemetry by saying it can provide a continuous location track as opposed to discrete start/stop endpoints.

We added this sentence to distinguish telemetry.

34-44: Too vague. Should introduce PIT tags, as they’re what this study used, in relation to other types of tagging.

We add an introduction on different types of tags.

48: Uncertainties? Parameters to estimate.

Revised.

54-57: Could at least mention nonlethal aging is possible, e.g. of fin rays: https://peerj.com/articles/1010/

Thanks. We mentioned nonlethal ageing and cited this reference.

57-58, “non-intrusive auxiliary measurements of tagged individuals, such as total length information, are recorded...” could be simplified to “length data can be recorded non-intrusively...”

Revised.

61: Age structure, not population structure

Revised.

*Methods*

104-110: How likely is it to go from 1 to 3 without going through 2 (i.e. through US waters)?

The time step of our models is one year. If one fish in MU1 in year 1, and in MU3 in year 2, it will contribute to movement probability from 1 to 3. There is no difference whether it goes from MU1 to 3 though MU2 or US waters. The actual trajectory is unknown, since this is not a radio tag. Only the end result can be observed.

113-114: Ref 33 does not use comparable data or apply a similar model. Block et al. (2005) used tags that record continuous tracks and created kernel density estimates for groups of fish (W/E spawners, size bins) to estimate probability of remaining in the west region or moving to the east. It’s not even a parametric model! Please cite another reference(s) where an age-structured tag-return model is used.

We cited other references that use similar models.

119: Clarify that pi are probabilities of movement.

Done.

123: Can you add any justification for this assumption? PIT tag retention rates are often high, so this is reasonable. I’d just add a reference, as specific to yellow perch and injection method (muscle, ventral) as possible.

We added a reference on PIT tag retention rates when applied to body cavity and dorsal muscle of yellow perch. Kaemingk MA, Weber MJ, McKenna PR, Brown ML. Effect of passive integrated transponder tag implantation site on tag retention, growth, and survival of two sizes of juvenile Bluegills and Yellow Perch. N Am J Fish Manag. 2011; 31: 726-732.

124: Clarify what is in Table 2: proportion of commercial catch sampled in region k and year fy. “Derived from”, or “equal to”?
A table listing the symbols in the model, clarifying which are estimated parameters versus fixed quantities, would be useful. For example, I believe the lambdas were calculated as in Table 2, not estimated.

The lambdas were assumed to be equal to the proportions of commercial catch sampled in region k and year fy, which was observed and fixed, not estimated. Symbols used in model equations include variables, estimated parameters and fixed quantities are listed in S Table 1 in Supporting Information.

144: “expected to be greater than 0.4”

Revised.

144-149: Curious why you didn’t restrict delta to be non-negative, since you have a strong expectation that M should not be less than 0.4. Although I see delta was estimated to be positive, so perhaps it doesn’t matter.

In this revision, we modeled M with a uniform prior over the interval (0.2, 2). Because we realized that there is a single M applied to all ages and areas so no need to model M hierarchically.

155-161: This seems odd. Instead of fixing 1 of 3 probabilities per region, why not use a likelihood for compositional data/parameters? Examples: multinomial, Dirichlet, Dirichlet-multinomial, logistic normal. Hyperpriors can easily be specified to make the prior probability of not moving slightly higher than moving.

In this revision, we set weakly informative Dirichlet priors with elemental gamma random variables gamma(1,1) for the three movement probabilities per region.

182: Would be useful to describe YR as “random effects by year”, as opposed to the fixed effects described on 175. Also would be useful to give a motivation, e.g. this reduces the effective number of parameters while still allowing movement to vary by year.

We added a sentence at the beginning of this subsection to give a motivation. “The setting of random effects reduced the effective number of parameters while still allowing movement to vary by year and age.”

195-199: Same as above.

Revised.

237-238: Instead of “relatively large samples”, please report the actual sample sizes.

“A total of 131,469 yellow perch were aged by otolith readings from 1989 to 2015.”

246-260: This section is titled “Likelihood” yet there is no data or likelihood described where the Pr(recap) and Pr(at large) are used. You probably meant to add a binomial or Bernoulli here...

We added description on likelihood function. “The tag recoveries by year of release (𝑡𝑦), region of release (𝑡𝑘), year of recovery (*fy*), region of recovery (*fk*) and length class at release (*l*) and tags that were never recovered were assumed to be multinomially distributed.” The likelihood function was provided in Equation 26.

261: Suggest “Model estimation” for the subtitle.

We changed the subtitle to “Model fitting and comparison”, because we talked about model fitting method, DIC, WAIC and LOO in this section.

267: I think using DIC for model selection is fine, but WAIC or LOO are generally preferred and can be calculated in JAGS models without too much extra work: https://link.springer.com/article/10.1007/s11222-016-9696-4.

Thanks for the reference. We computed WAIC and LOO using R loo package.

*Results*

275-277: Calling the estimated year and age effects on movement rates “substantial” is a stretch. If you added CIs to Fig. 5 my guess is there would be quite a bit of overlap. The posteriors in in Figs. 2-4 are almost completely overlapping with no discernible differences apart from the two examples you highlight later (2012 more 3 -> 3, 2011 more 2 -> 3). I think this sentence should be dropped or modified.

We dropped this sentence.

277-281: Using random effects vs. fixed effects terminology consistently to describe the difference between models YR vs. YS and AR vs. AS would make the paper clearer.

Done.

297-300: Suggest adding that most year and age effects were not pronounced, except for the two examples you highlight.

We changed this section to “Although year and age-variations in movement probabilities derived from Model AY (Figs 3 and 4) were not substantial, there were obvious differences on posterior medians in some years and age classes, for example, posterior median of movement probability $\pi_{1,1}$ in 2013 was 0.90 that was 3-5% greater than $\pi_{1,1}$ in other years; posterior median of movement probability $\pi_{2,3}$ in 2011 was 0.70 that was 3-5% greater than $\pi_{2,3}$ in other years; posterior medians of movement probability $\pi_{3,3}$ in 2012 and 2014 were 0.65 and 0.63 respectively that were 3-9% greater than $\pi_{3,3}$ in other years; posterior median of movement probability $\pi_{1,2}$ at age-4 was 0.13 that was 30-184% greater than $\pi_{1,2}$ at the rest age classes; posterior median of movement probability $\pi_{2,3}$ at age-4 was 0.72 that was 2-10% greater than $\pi_{2,3}$ at the rest age classes; posterior median of movement probability $\pi_{3,2}$ at age-4 was 0.33 that was 29-58% greater than $\pi_{3,2}$ at the rest age classes. Furthermore, Model AY performed best with the smallest DIC, WAIC and LOO values (Table 3), so it was recommended to better understand year-variations in fish movement probabilities, which might be linked to environmental changes. Model AR was recommended when integrating short-term tagging data with long-term fisheries data.”

312-322: Could be summarized more briefly—across all ages and years most yellow perch in MU1 stayed in MU1. 316-318 can be cut, does not add any new information.

Done.

343-343: M = 1.3 implies that delta = 1.18. If nu = 0.1, your estimate of delta is 11.8 standard deviations from the mean.

In this revision, we modeled M with a uniform prior over the interval (0.2, 2). Because we realized that there is a single M applied to all ages and areas so no need to model M hierarchically.

*Discussion*

374-377: Should report the estimate of M with 95% CI here, instead of just stating it was greater than 0.4. Also, M = 1.3 is quite a bit higher than 0.4. Are estimates of F from bycatch or U.S. harvest available to account for a substantial portion of this difference? Otherwise, that is a lot of emigration, tag loss, and/or tagging mortality.

We reported the estimate of M with 95% CI here, and obtained estimates of commercial trap net F and angler F from the YPTG stock assessment report. “The natural mortality rate is estimated with a posterior median of 1.36 and a 95% credible interval of (1.19, 1.53), which is much larger than the 0.4 used by the YPTG. This could have been caused by many factors, including fishing activities other than the Ontario commercial gillnet fishery, bycatch, migration outside of the study area, tag shedding and tag-induced mortality. Point estimates of commercial trap net and angler fishing mortality rates in the U.S. waters estimated from the YPTG stock assessment model [49] are plotted in S Fig 6 in Supporting Information. The sum of these two fishing mortality rates is large in MU2 in 2014 and 2015 (> 0.4). If a large proportion of the estimated natural mortality is actually fishing mortality from U.S. waters, our models is overestimating the natural mortality of young (pre-selected) fish. Additional information could be collected to account for other factors; for example, double-tagging experiments have been widely used to quantify tag-shedding [54,56–58].”

378: I’m not sure that additional information *needs* to be collected to account for these factors... your model is useful as is. Additional information *could be* collected.

Changed to “could be”.

397-398: But this was true in all years, not just 2012.

We rewrote this sentence to “The high annual average surface temperature and low ice cover associated with high winter NAO index in 2012 indicate a relatively short, warm winter”. The annual average surface temperature, ice cover and winter NAO index are plotted in Fig 6.

428: Region was not a random effect in the model

Yes, we deleted “region”.

*Figures*

Not absolutely necessary, but the multi-panel figures 2-5 and 7 would look better with reduced margins and shared axes.

We reduced margins and shared axes.

Figs. 2-5: Because the year and age effects are additive (the effect of year is the same across ages), I think these could be improved by displaying one set focused mean movement (comparing priors and posteriors), one set on year effects (i.e. Fig. 5, removing ages and adding credible intervals instead), and one set focused on age effects (i.e. Figs. 2-4, removing years). CIs should be added so the reader can see the degree of age and year effects.

Done. Please see our new Figs 2, 3 and 4.

Fig. 5 has different y-axes for each panel. Keeping the y-axes consistent from 0-1 would make it easier to digest where the fish are moving.

Done.

Figs. 5 and 7 do not have estimates of uncertainty, and therefore it is impossible to know what degree the annual variations are significant. CIs should be added.

We added CIs on Fig 5, which are new Figs 3 and 4 in this revision. Fig 7 (new Fig 6 in this revision) is environmental variables, and point values.

Fig. S1: A gradient color scale may make this easier to digest (i.e. adjacent length bins should be more similar in color).

Done.
